# Immune Checkpoint Inhibitors in Colorectal Cancer: Challenges and Future Prospects

**DOI:** 10.3390/biomedicines9091075

**Published:** 2021-08-24

**Authors:** Shima Makaremi, Zahra Asadzadeh, Nima Hemmat, Amir Baghbanzadeh, Alessandro Sgambato, Farid Ghorbaninezhad, Hossein Safarpour, Antonella Argentiero, Oronzo Brunetti, Renato Bernardini, Nicola Silvestris, Behzad Baradaran

**Affiliations:** 1 Department of Immunology & Microbiology, School of Medicine, Arak University of Medical Sciences, Arak 3848176941, Iran; sh.makaremi73@gmail.com; 2 Immunology Research Center, Tabriz University of Medical Sciences, Tabriz 5166/15731, Iran; Zahraasadzadeh2834@gmail.com (Z.A.); nima.hemmat1995@gmail.com (N.H.); amirbaghbanzadeh@gmail.com (A.B.); ghorbaninezhadfarid@gmail.com (F.G.); 3 Istituto di Ricovero e Cura a Carattere Scientifico Centro di Riferimento Oncologico della Basilicata (IRCCS-CROB), 5972362 Rome, Italy; alessandro.sgambato@crob.it; 4 Area of Pathology, Department of Woman and Child Health and Public Health, Fondazione Policlinico Universitario A. Gemelli-IRCCS, 5972362 Rome, Italy; 5 Department of Immunology, School of Medicine, Tabriz University of Medical Sciences, Tabriz 5166/15731, Iran; 6 Cellular & Molecular Research Center, Birjand University of Medical Sciences, Birjand 9717853577, Iran; safarpour701@yahoo.com; 7 IRCCS Istituto Tumori “Giovanni Paolo II” of Bari, 70124 Bari, Italy; argentieroantonella@gmail.com (A.A.); dr.oronzo.brunetti1983@gmail.com (O.B.); 8 Department of Biomedical and Biotechnological Sciences, University of Catania, Via S. Sofia 97, 95121 Catania, Italy; bernardi@unict.it; 9 Department of Biomedical Sciences and Human Oncology (DIMO), University of Bari, 70124 Bari, Italy; 10 Pharmaceutical Analysis Research Center, Tabriz University of Medical Sciences, Tabriz 5166/15731, Iran

**Keywords:** cancer immunotherapy, immune checkpoints, colorectal cancer, monoclonal antibodies (mAbs)

## Abstract

Immunotherapy is a new pillar of cancer therapy that provides novel opportunities to treat solid tumors. In this context, the development of new drugs targeting immune checkpoints is considered a promising approach in colorectal cancer (CRC) treatment because it can be induce specific and durable anti-cancer effects. Despite many advances in the immunotherapy of CRC, there are still limitations and obstacles to successful treatment. The immunosuppressive function of the tumor microenvironment (TME) is one of the causes of poor response to treatment in CRC patients. For this reason, checkpoint-blocking antibodies have shown promising outcomes in CRC patients by blocking inhibitory immune checkpoints and enhancing immune responses against tumors. This review summarizes recent advances in immune checkpoint inhibitors (ICIs), such as CTLA-4, PD-1, PD-L1, LAG-3, and TIM-3 in CRC, and it discusses various therapeutic strategies with ICIs, including the double blockade of ICIs, combination therapy of ICIs with other immunotherapies, and conventional treatments. This review also delineates a new hopeful path in the combination of anti-PD-1/anti-PD-L1 with other ICIs such as anti-CTLA-4, anti-LAG-3, and anti-TIM-3 for CRC treatment.

## 1. Introduction

Colorectal cancer (CRC) is one of the prevalent malignancies with a high mortality rate worldwide [1,2,3]. CRC incidence is increasing, and it is estimated that the number of CRC patients will reach 2.5 million by 2035 [4]. CRC is invasive cancer, the initiation and progression of which involve both hereditary and environmental factors [4]. The current therapeutic approach at the early stages of CRC is surgery, followed by radiotherapy and chemotherapy [5]. These common treatments may give rise to several challenges, such as their side effects, and are often associated with the development of drug resistance [5,6]. In order to overcome these obstacles, other therapeutic approaches and common therapies aiming to achieve better results are required. Among the new treatment options, immune checkpoint inhibitors (ICIs) are promising approaches in cancer therapy and have been successfully exploited for the treatment of melanoma, renal cell carcinoma (RCC), bladder cancer, lung cancer, and CRC [7,8,9,10,11].

The immune system has a significant role in halting tumor cell development. As such, immunotherapy, such as using monoclonal antibodies (mAbs) that target immune checkpoints, could significantly impact the treatment process of various malignancies [12]. Immune checkpoint molecules and their cell surface receptors have essential roles in modulating the immune system. Under normal conditions, immune checkpoints can maintain tolerance by sending inhibitory signals to T cells. Therefore, mAbs targeting immune checkpoints enhance T cells’ antitumor immune response and improve antitumor defense [7,13]. Cytotoxic T-lymphocyte-associated antigen 4 (CTLA-4), programmed death 1 (PD-1), and its ligand (PD-L1), as well as the B7 family of immune checkpoints, could be considered to be the main immunotherapy targets to inhibit tumor growth in a variety of cancers [14,15,16].

This review analyzes the biological aspect and presents preclinical and clinical data of CTLA-4, PD-1/PD-L1, and other new inhibitory immune checkpoints such as lymphocyte-activation gene 3 (LAG-3) and T-cell immunoglobulin- and mucin domain-3-containing molecule 3 (TIM-3) in the induction and progression of CRC. In addition, other potential therapeutic approaches, such as combining ICIs and radiotherapy or chemotherapy, are considered.

## 2. Search Strategy and Selection Criteria

We searched PubMed, Web of Science, Google Scholar, and conference/congress paper using the search terms “colorectal cancer” or “CRC” along with these terms “immune checkpoint”, “CTLA-4”, “PD-1”, “PD-L1”, “LAG-3”, “TIM-3”, and “monoclonal antibodies (mAbs)” in combination with specific mAbs for each immune checkpoint. By using this strategy, we carried out the screening process and selected relevant papers.

## 3. The Immune Microenvironment in Colorectal Cancer (CRC)

One of the most important functional aspects of the immune system is detecting and destroying tumor cells. This phenomenon is called immune surveillance, which Paul Ehrlich proposed. When the immune system is unable to correctly identify or eradicate tumor cells, it contributes to the expanded proliferation of malignant cells and the induction of cancer [17]. The cancer immunoediting concept comprises three phases: elimination, equilibrium, and escape. In the first phase, immune system components such as natural killer cells (NK cells), as the innate arms of the immune system, together with T CD4^+^ and T CD8^+^ cells, as the adaptive arm of the immune system, can succeed in eradicating tumor cells. The equilibrium phase is known as the state of balance between immune and tumor cells. In this phase, tumor growth could be restricted, and the survived tumor cells can evade the immune system. In the last phase, the escape phase, tumor cells escape immune system control and can grow unlimitedly [18,19].

Maintaining immune homeostasis is considered a fundamental property of the normal function of the immune system [20]. As a result of tumor development, immune homeostasis is disrupted, leading to the impaired function of immune cells within the tumor microenvironment (TME) [21]. The TME of CRC has a significant and decisive effect on tumor growth and progression. CRC is one of the most prevalent solid tumors and manifests with heterogeneous tumor masses resistant to immune system responses. The local microenvironment of solid tumors masses is surrounded by various types of cells, including innate immune cells (NK cells, tumor-associated macrophages (TAMs), dendritic cells (DCs), and mast cells (MCs)), as well as adaptive immune cells such as T and B cells [22]. Moreover, the CRC microenvironment consists of different types of diseased cells such as malignant epithelial cells, cancer-associated fibroblasts (CAFs), mesenchymal cells, endothelial cells, and cancer stem cells (CSCs) [23,24,25].

### 3.1. Immune Cells Involved in Tumor Suppression in the TME

Tumor-infiltrating immune cells within the TME are composed of DCs, macrophages, NK cells, T cells, B cells, and other cells of the innate immune system [22,26]. The innate immune system is responsible for the primary defense against tumor cells. This arm of the immune system encompasses various cells and components that can detect tumor antigens. DCs, macrophages, and NK cells have crucial roles in stimulating the adaptive immune system against tumor cells [27]. Furthermore, NK cells are critical in the restriction of tumor cell growth. NK cells express some receptors on their surface that recognize tumor antigens and exert cytotoxic activity against tumor cells. According to available studies, an abundance of infiltrating NK cells and CD8^+^ T cells is correlated with a better prognosis in CRC [27,28].

The type and density of these cells in the TME of CRC could greatly influence tumor progression [29]. In this context, as the parts of the cellular adaptive immune response, T helper cells (CD4^+^ T cells) and cytotoxic T lymphocytes (CTLs) inhibit tumor growth and development, while regulatory T cells (Tregs) promote tumor progression. CTLs and CD8^+^ T cells are considered major components with antitumor activity. CTLs display a cytotoxic effect and directly kill tumor cells by recognizing tumor antigen peptides expressed by antigen-presenting cells (APCs) like DCs [30,31]. In addition to CTLs, CD4^+^ helper T cells present in the TME are involved in activating CTLs against tumor cells. Additionally, CD4^+^ helper T cells play an essential role in maintaining CTL antitumor response [31]. Long-term interaction between CTLs and antigens could lead to the exhaustion of CTLs, by which these cells lose their efficiency and functions. Tumor cells suppress the immune response by inducing the exhaustion of CTLs. The exhaustion of CTLs is a mechanism that occurs in the TME, suppressing the immune response of CTLs against tumor cells through the expression of inhibitory receptors such as *PD-1*, *CTLA-4*, and *LAG-3* [32,33]. Therefore, blocking these inhibitory receptors by mAbs could prevent CTL exhaustion and reinvigorate the antitumor function of CTLs.

### 3.2. Immune Cells of TME Involved in Tumor Progression

The progression and evasion of tumor cells in the TME is mediated by several types of immunosuppressive cells such as Tregs, TAMs, myeloid-derived suppressor cells (MDSCs), and CAFs. Many studies have demonstrated that the presence of an immunosuppressive cell’s enrichment in the TME could contribute to tumor progression and expansion [23,34,35,36,37]. Regulatory T cells comprise another substantial component of TME that favor tumors development and promote tumor growth. The presence of Tregs as immunosuppressive cells in the TME could favor the evasion and proliferation of tumor cells. FoxP3^+^ Tregs express inhibitory immune checkpoints (*CTLA-4*, *PD-1*, and *LAG-3*) and produce immunosuppressive cytokines such as IL-10, TGF-β, and IL-35 [36]. One of the most likely explanations for Treg accumulation in the CRC microenvironment is the IL-33/ST2 pathway, which plays a fundamental role in Tregs’ activation and function, as well as in TME remodeling [38]. Additionally, FoxP3^+^ Tregs may be considered prognostic biomarkers at the early stage of CRC [39].

Thus far, two phenotypes of macrophages have been identified—the M1 and M2 types, which are categorized based on their functions. M1 phenotypes or classically activated macrophages are characterized by antitumor activity and could produce type 1 cytokines. On the other hand, M2 phenotypes, termed alternatively activated macrophages, exert tumorigenesis function and enhance tumor growth and metastasis [34]. TAMs, similar to M2 macrophages, have immunosuppressive activity and, within TME, support the invasion and metastasis of solid tumors, particularly CRC [40]. Furthermore, TAMs can suppress the immune response by inhibiting M1 macrophages and their antitumor activity, as well as by disrupting T cell functions [35].

Together with Tregs and TAMs, MDSCs are also present in the TME and cooperate in supporting the tumor proliferation, angiogenesis, metastasis, and escape of cancer cells. MDSCs are a heterogeneous group of cells derived from the myeloid lineage of the bone marrow [41]. Within the TME, MDSCs can promote tumorigenesis and the disruption of T cell antitumor activity through the induction of several immunosuppressive mediators such as prostaglandin E2 (PGE2), transforming growth factor (TGF-β), and IL-10, as well as the enhancement of the expression of arginase-1 (ARG1) and inducible nitric oxide synthase (iNOS) [42]. An increased number of MDSCs has been found to be associated with an increased risk of metastasis in CRC [43,44].

CAFs are heterogeneous non-immune cells located in the TME that mediate tumor progression and metastasis. *TGF-β* and *p53* mutations are the main factors in converting normal fibroblast cells into CAFs in cancerous conditions. CAFs could support the invasion and progression of tumors by interacting with other immunosuppressive cells (Tregs, TAMs, and MDSCs) in the TME. Additionally, they could exert negative effects on CTLs’ and NK cells’ antitumor activity [45]. According to evidence, the levels of CAFs e correlate with the levels of TGF-β in the CRC microenvironment [46,47].

**Figure 1:**
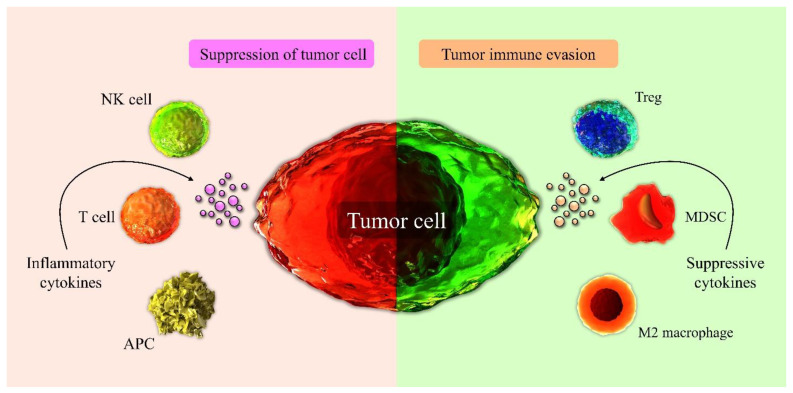
The tumor microenvironment is involved in tumor progression. In the TME, the populations of NK cells, APCs, and T cells exert an antitumor response and can lead to the suppression of tumor growth by producing inflammatory cytokines and causing the direct destruction of tumor cells. Conversely, the immunosuppressive microenvironment composed of inhibitory cells like Tregs, MDSCs, and M2 macrophages contributes to tumor development by attenuating the activity of antitumor immune cells.

Interestingly, it has been reported that one of the most important factors that affects immune cells and the TME in CRC is the host microbiome. The composition and diversity of various gut microbiome species affect the immune cells’ response against tumor cells. In this context, the role of bacterial species is more critical than other microbiome populations. Some bacterial species, such as *Fusobacterium nucleatum*, could promote tumor progression by modulating the immune system. Moreover, gut microbiome components could be involved in response to chemotherapy and immunotherapy, and they may affect the effectiveness of these treatments [19,29]. A better understanding of the interaction between the immune system and cancer cells in the TME could allow for the better control of tumor growth and progression. Within this context, a better understanding of the immune checkpoint activity that plays a pivotal role in regulating T cell effector functions is essential to identify practical and valuable targets in solid tumors such as CRC. Several immunotherapy strategies are currently used to enhance the immune response against CRC cells. Therapeutic approaches targeting these inhibitory receptors and blocking immune checkpoints are able to support T cell activity and promote T cells’ antitumor immune reactions within the CRC microenvironment [48]. The significant effects of immune cell population in the TME are shown in Figure 1.

## 4. Immune Checkpoint Molecules

The immunoregulatory cells (Treg, MDSCs, and M2 macrophages) and cytokines (IL-10 and TGF-β) possess the ability to control and modulate T cell function through the release of molecules able to activate specific inhibitory immune checkpoints [49,50,51]. However, tumor cells and other cells in the TME can also express these inhibitory receptors’ ligands and, therefore, activate these inhibitory checkpoints, thus impairing T cells’ activity [13]. In this way, activating inhibitory immune checkpoints may disrupt the proliferation of CTLs in CRC and reduce the immune response against cancer [52]. This section describes the biological effects and functions of CTLA-4, PD-1/PD-L1, LAG-3, and TIM-3 as inhibitory immune checkpoints.

### 4.1. CTLA-4

Cytotoxic T-lymphocyte-associated protein-4 (CTLA-4; CD152) is one of the inhibitory immune checkpoints expressed on activated T cells and Treg cells. Together with CD28, CTLA-4 plays a critical role in the initial activation and subsequent control of cellular immunity. Whereas CD28 primarily activates T cell processes, CTLA-4 inhibits them. CTLA-4, as a type 1 transmembrane glycoprotein, belongs to the immunoglobulin superfamily. Its gene is located on band q33 of chromosome 2 and encodes for a protein of 223 amino acids [53]. CTLA-4 acts as an inhibitory receptor by binding to its ligands, CD80 and CD86, on the APCs, and it has a higher affinity for CD80 and CD86 ligands in comparison with CD28 [54]. The activation of T cells requires two different signals: the first signal depends on T-cell receptor (TCR) recognition of major histocompatibility complex (MHC) class I or class II molecules loaded with antigenic peptides on the surface of APCs, and the second signal consists of an interaction between co-stimulatory receptors such as CD28 on T cell and its ligands, CD80 (B7-1) and CD86 (B7-2), on APCs [55,56]. CD28/B7 binding is necessary for the full activation of naïve T cells, leading to increased IL-2 production and allowing T cells to proliferate and differentiate [57]. T cell activation could be regulated by the co-stimulatory (CD28/B7) or co-inhibitory (CTLA-4/B7) function of immune checkpoint receptors. Thus, signaling via co-stimulatory receptors, such as CD28, is required for T cell activation. On the other hand, signaling via co-inhibitory receptors, such as CTLA-4, is a negative signal and inhibits T cell proliferation [58]. Notably, in naïve T cells, CTLA-4 is not expressed on the cell surface and has an intracellular location. CTLA-4 could be induced on T cells following activation through TCR/CD28 co-stimulation and inhibits T cells’ activation by blocking CD28/B7 signals. This function of CTLA-4 in moderate T cell activation is essential in preventing autoimmunity [59]. CTLA-4 is also constitutively expressed on CD4^+^ Foxp3^+^ Tregs and is required for Tregs’ regulatory function. [60]. CTLA-4 suppresses T-cell function with different pathways, such as promoting inhibitory cytokines and indoleamine 2, 3-dioxygenase (IDO) [61]. Accordingly, targeting CTLA-4 is a proper candidate for immunotherapy and the treatment of various types of malignancies such as CRC.

### 4.2. PD-1/PD-L1

The surface receptor, programmed cell death-1 (PD-1, PDCD1), as a negative immune checkpoint, was first discovered on murine T cell hybridoma [62]. This checkpoint is involved in suppressing T cell antitumor functions and causes the escape of tumor cells from the immune response. Like CTLA-4, PD-1 (CD279) belongs to the CD28 immunoglobulin family, a subgroup of inhibitory immune checkpoints that is constitutively expressed on the T cell population [63]. The PD-1 gene is located on chromosome 2q37, and this gene encodes a protein of 288 amino acids with a 55 kDa molecular weight. Its monomer structure consists of three parts: an extracellular N-terminal IgV-like domain, a transmembrane domain, and a cytoplasmic domain [64]. The cytoplasmic tail of PD-1 has two tyrosine-based motifs: an immunoreceptor tyrosine-based inhibitory motif (ITIM) and an immunoreceptor tyrosine-based switch motif (ITSM). PD-1 inhibits the activation of T cells by recruiting protein tyrosine phosphatase SHP-2, which interacts with ITSM motifs [65,66]. In addition to T cells, other cell types such as B cells, monocytes, and DCs express PD-1 [67]. Of note, the expression of PD-1 can be induced following T cell activation by TCR complex stimulation and the secretion of multiple cytokines, such as IL-2, IL-7, IL-15, and IL-21 [68]. PD-1 performs its suppressive function by interacting with its ligands, PD-L1 (B7-H1) and PD-L2 (B7-DC). PD-L1, as a cell surface glycoprotein, is expressed on a variety of cell types, such as T cells, B cells, endothelial cells, and tumor cells [69,70]. PD-L2, as a second ligand for PD-1, has a more limited expression than PD-L1. Indeed, only APCs and non-hematopoietic tissues can express PD-L2 on their surfaces [64]. Interferon (IFN)-γ can significantly enhance PD-L1 expression [65]. Overall, the PD-1 pathway has a crucial role in maintaining peripheral tolerance in normal conditions to prevent autoimmune diseases; however, in the TME, this pathway leads to the escape of tumor cells from immune response via the inhibition of CTL activation [64,71]. Additionally, the expression of PD-L1 on tumor cells is related to the exhaustion of T cells; therefore, blocking the PD-1 pathway has been demonstrated to be a successful approach for the treatment of different types of cancers including non-small cell lung cancer (NSCLC), melanoma, breast, RCC, and CRC [70,72,73,74,75].

### 4.3. LAG-3

Lymphocyte-activation gene 3 (LAG-3, CD223) was first identified in the 1990s as a member of the immunoglobulin superfamily on a subset of NK cells, and it acts as a negative checkpoint on T lymphocytes [76,77]. The LAG-3 protein consists of 498 amino acids, and its gene is located on human chromosome 12 [78]. Structurally, LAG-3 contains four extracellular Ig-like domains (D1–D4) and the extracellular (EC) region [79,80]. LAG-3 is an inhibitory receptor that is mainly expressed by CD4^+^ T cells and tempers their homeostatic expansion. LAG-3 and CD4 are structurally similar, and this homology has made MHCII a ligand for LAG-3. The LAG-3 utilizes its D1 domain for binding to MHCII with a higher affinity than CD4 [78,80]. This immune checkpoint is expressed on activated CD4^+^ T and CD8^+^ T cells, Tregs, NK cells, invariant NK T cells, B cells, and TILs. Additionally, the interaction of immature APCs, such as DCs, with LAG-3^+^ Tregs causes the inhibition of their maturation [81,82,83]. LAG-3, as an inhibitory receptor, regulates T-cell functions and plays a crucial role in preventing autoimmune disorders. On the other hand, the expression of LAG-3 in the TME could inhibit T cell function and promote tumoral immune escape [80,84]. Moreover, there is a significant association between the upregulation of LAG-3 and T cell exhaustion [85]. LSECtin (liver sinusoidal endothelial cell lectin) and galectin-3 are the secondary ligands for LAG-3 [78]. The interaction between galectin-3 and LAG-3 could inhibit CD8^+^ T cell function in the TME. LSECtin, as a member of the DC-SIGN family, is expressed on liver and melanoma cells and can bind LAG-3, thus promoting tumor progression. Additionally, it is correlated with the inhibition of IFN-γ secretion from T cells, ultimately leading to tumor escape [81,86]. CRC is a cancer with high expression of LAG-3, so targeting LAG-3 may be an excellent therapeutic approach to treat such solid tumors [87].

### 4.4. TIM-3

T-cell immunoglobulin- and mucin domain-3-containing molecule 3 (TIM-3) is an immune regulatory molecule and has a significant role in immune tolerance [88]. TIM-3, known as Hepatitis A virus cellular receptor 2 (HAVCR2), is a member of the TIM gene family, and its gene is located on human chromosome 5q33.2 [89]. TIM-3, as a type 1 membrane protein, is formed by 301 amino acids with four components: an extracellular domain, a mucin domain, a single transmembrane region, and a C-terminal cytoplasmic tail [88,90]. TIM-3 is mainly represented on the surface of CD4^+^ T helper 1 and CD8^+^ T cytotoxic cells. Furthermore, it can be expressed on Tregs, B cells, and innate immune cells like DCs, macrophages, and NK cells [88,89]. The ligand for TIM-3 is galectin-9 (LGALS9), which belongs to the galectin family of lectins [91]. Galectin 9 can be expressed on different cell types, especially on the cells of lymphatic organs such as the spleen, small intestine, thymus, liver, kidney, colon, placenta, and pancreas [92,93]. The binding of TIM-3 to galectin-9 on T helper cells can lead to the apoptosis of Th1 cells via the release of Bat3 from the cytoplasmic tail of TIM-3 [93,94]. Additionally, this interaction inhibits IFN-γ production in Th1 cells [95]. Phosphatidylserine (PtdSer), high mobility group protein B1 (HMGB1), and carcinoembryonic antigen cell adhesion molecule 1 (Ceacam-1) are reported to be the other ligands for TIM-3 [94,96]. The binding of PtdSer to the FG and CC′ loops of the TIM-3 IgV domain may improve antigen cross-presentation in TIM-3^+^ DCs [88]. HMGB1 is the third ligand for tumor-infiltrating DCs that expresses a high level of TIM-3. TIM-3 can also prevent innate immune activation by inhibiting the binding of HMGB1 to nucleic acids released from dying tumor cells; therefore, it has a negative role in antitumor response [97,98]. CEACAM1, another ligand for TIM-3, can be expressed on activated T cells and, in collaboration with TIM-3, inhibits T cell responses [99]. CEACAM1 interacts with the IgV domain of TIM-3 and restrains the TCR signaling by releasing Bat3. The cis or trans interactions of TIM-3 with CEACAM1 prevent T cell immune response [97]. Moreover, the expression of TIM-3 and CEACAM1 is augmented on circulating CD8^+^ T cells in CRC patients, which is mainly associated with T cell exhaustion, especially CD8^+^ T cells [99,100]. Due to the incremented expression of TIM-3^+^ PD-1^+^ CD8^+^ T cells in the blood and tumor tissue of CRC patients [100], TIM-3 can be considered a valuable therapeutic target in CRC [101].

## 5. Immunotherapy with Immune Checkpoint Inhibitors (ICIs)

**Figure 2:**
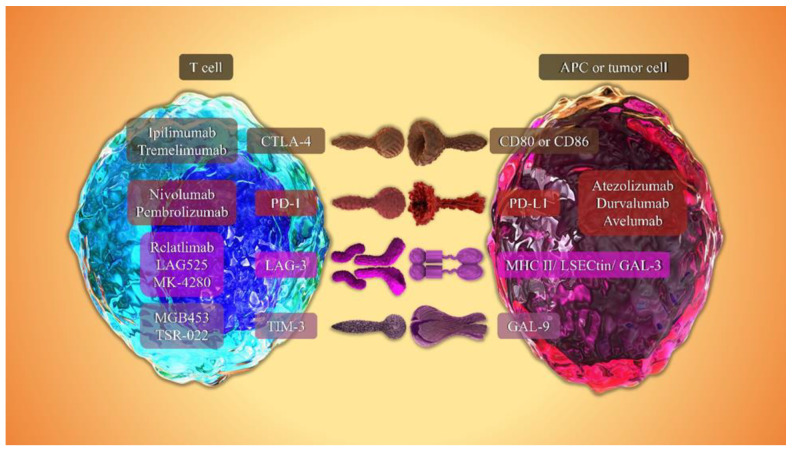
The overview of monoclonal antibodies against immune checkpoints and their ligands. Cytotoxic T lymphocyte antigen 4 (CTLA-4), programmed cell death 1 (PD-1), programmed cell death 1 ligand 1 (PD-L1), lymphocyte-activation gene 3 (LAG-3), T-cell immunoglobulin- and mucin domain-3-containing molecule 3 (TIM-3), LSECtin (liver sinusoidal endothelial cell lectin), galectin-3 (GAL-3), and galectin-9 (GAL-9).

**Figure 3:**
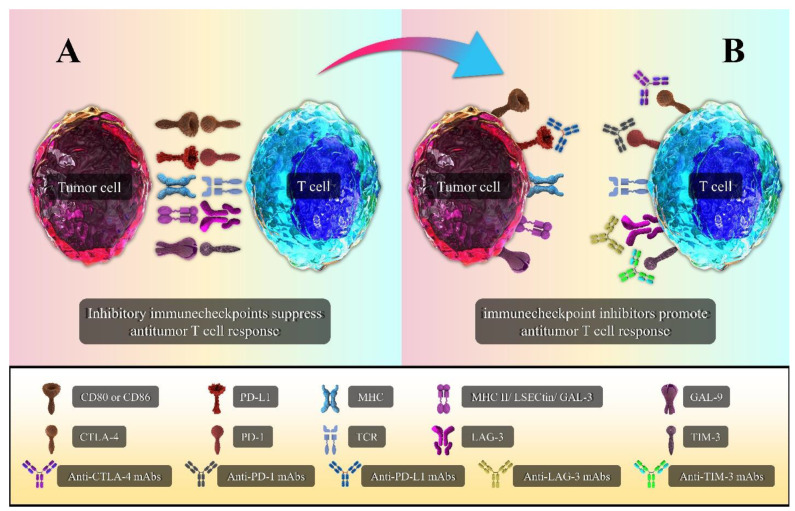
Targets of immune checkpoint inhibitors. (**A**) The interaction between inhibitory immune checkpoints with their ligands inhibits T cells’ antitumor function. (**B**) Engaging monoclonal antibodies (mAbs) with inhibitory immune checkpoints enhances T cells’ antitumor response by preventing T cell suppression.

Conventional cancer therapies such as surgery, radiation, and chemotherapy have dominant use in many cancers. These therapies can mostly be effective at the early stage of cancer progression but might cause resistance and have severe side effects [102,103]. Additionally, in many patients, the evasion of tumor cells from immune surveillance plays an essential role in the process of tumor development and progression. Therefore, novel approaches are required to overcome these problems to achieve the proper cancer treatment. Immunotherapy with ICIs is a novel approved approach for the treatment of malignancies like melanoma, NSCLC, and CRC [104,105]. Considering the vital role of T cells in the destruction of cancer cells, ICIs can be beneficial by promoting T cell’s responses during immune system response against a tumor [106]. Various studies using ICIs in CRC (individually or combination therapy) have demonstrated a positive effect of these drugs for the treatment of CRC patients, but they still need further evaluation [104,107,108]. In recent decades, cancer immunotherapy using mAbs that target immune checkpoints has come to be considered one of the major immunotherapeutic approaches. The blocking of immune checkpoints reactivates T cell functions, especially those of CTLs, contributing to continuing antitumor functions and improving the host immune response against cancer. Targeting immune checkpoints can improve outcomes in several types of cancers such as lung cancer, liver, melanoma, ovarian, and prostate cancers [109]. The most well-known mAbs that target inhibitory checkpoints are Ipilimumab and Tremelimumab, which act as anti-CTLA-4 agents; Nivolumab and Pembrolizumab, which act as anti-PD-1 agents; and besides Atezolizumab and Durvalumab, which are anti-PD-L1 mAbs. This section discusses the role of anti-CTLA-4 and anti-PD-1/PD-L1 mAbs, as well as novel immune checkpoints (anti-LAG-3 and anti-TIM-3), in CRC. The most important ICIs and their interactions with their ligands are illustrated in Figure 2 and Figure 3, respectively.

### 5.1. Anti-CTLA-4

A blockade of CTLA-4 with mAbs is a promising anticancer strategy, promoting antitumor response by enhancing T cell activation [110]. Anti-CTLA-4 antibodies could attach to their receptors (CTLA-4/B7) on the surface of T cells, thus improving T cells’ antitumor function through prolonging T cell activity [111]. Treg cells, as a suppressive component of the immune system, constitutively expresses CTLA-4; therefore, the use of anti-CTLA-4 mAbs may enhance antitumor responses by reducing Treg cell function [105]. An immune checkpoint blockade provides a promising therapeutic approach for patients with mismatch repair deficient (dMMR)/microsatellite instability-high (MSI-H) mCRC [112]. The anti-CTLA-4 mAb, Ipilimumab, is a fully human IgG1 approved by the FDA for melanoma cancer in 2011 [113]. As a specific CTLA-4 blockade, Ipilimumab can reinforce T cells’ antitumor responses by preventing CTLA-4 with B7 and allowing CD28 to bind to B7, resulting in continuous T cell activation [114]. This immune checkpoint blockade combined with Nivolumab, an anti-PD-L1 mAb, showed a high antitumor response in patients with dMMR/MSI-H mCRC [115]. Tremelimumab is another fully human IgG2 immunoglobulin anti-CTLA-4 mAb that is under investigation to treat patients with solid tumors [116]. In a phase II clinical study, this mAb was not effective alone in patients with refractory metastatic CRC [117]. However, studies have shown the beneficial efficacy of Tremelimumab in patients with advanced hepatocellular carcinoma [118,119]. Moreover, the results of a phase II study showed that the combination of Tremelimumab (anti-CTLA-4) with Durvalumab (anti-PD-L1) could increase the overall survival (OS) of patients with advanced refractory CRC [120]. Therefore, the combination of anti-CTLA-4 with other ICIs such as anti-PD-L1 may be more effective than targeting anti-CTLA-4 as a single agent in CRC.

### 5.2. Anti-PD-1

The PD-1/PD-L1 cascade, as an inhibitory pathway, has an efficient role in modulating T-cell activation and is responsible for maintaining peripheral tolerance [121]. Blockade of this pathway via mAbs could promote T cell’s antitumor activity [66]. Notably, PD-1 expression increases on the surface of T CD8^+^ cells in the CRC TME. Therefore, the blockade of PD-1 can be a practical approach for treating CRC [122]. The two known FDA-approved anti-PD-1 mAbs are Nivolumab and Pembrolizumab [123]. Nivolumab has firstly received FDA approval for melanoma patients with advanced disease in 2014 [124]. Nivolumab is a fully-humanized immunoglobulin G4 (IgG4) anti-PD-1 monoclonal antibody that is FDA-approved to treat various cancers such as melanoma, NSCLC, RCC, and Hodgkin’s lymphoma [65]. A study considering the use of Nivolumab in patients with dMMR/MSI-H metastatic CRC showed durable responses in patients with experience of previous treatments. In this trial, patients who were treated with Nivolumab received the intravenous administration of 3 mg/kg of Nivolumab every 2 weeks. The administration continued until disease progression, death, unacceptable toxic effects, withdrawal of consent, or the end of the study. Of note, 23 patients of 74 (31%) attained an objective response, and 51 patients (69%) demonstrated controllable disease for 12 months or more in a median follow-up of 12 months [125]. Moreover, phase I and II clinical trials showed the positive effects of Nivolumab and other ICI in the MSI-H mCRC therapy [126]. Another anti-PD-1 mAb is Pembrolizumab, which is also an FDA-approved fully-humanized monoclonal IgG4 antibody [127,128]. Pembrolizumab was investigated with napabucasin in patients with MSI-H/MSS mCRC. The results of a phase I/II trial demonstrated the efficacy of Pembrolizumab (200 mg every 3 weeks) with napabucasin (240–480 mg twice daily) against MSI-H/MSS mCRC [129]. Another study was performed to appraise the effect of Pembrolizumab on colorectal patients that expressed PD-L1, and it confirmed the suitability of this drug in PD-L1-positive CRC patients [130]. Furthermore, targeting PD-1 immune checkpoints with a combination of anti-PD-1 mAbs (Nivolumab with low-dose Ipilimumab) might represent a promising therapeutic strategy in patients with previously treated MSI-H/dMMR mCRC [131].

### 5.3. Anti-PD-L1

PD-L1 has been considered a component of the PD-1/PD-L1 pathway that suppresses a T cell’s antitumor function by binding to its ligand, PD-1. In addition to PD-1, PD-L1 can be targeted with mAbs to prevent T cell signaling attenuation [70,132]. Anti-PD-L1 mAbs, including Atezolizumab, Durvalumab, and Avelumab, are used to treat melanoma, NSCLC, and RCC, respectively [123,133]. Atezolizumab is an anti-PD-L1 humanized IgG1 mAb that displays therapeutic efficacy in some cancers such as metastatic urothelial cancer and lung cancer [132,134]. A phase Ib investigation on the efficacy of Atezolizumab in combination with Bevacizumab (anti-VEGF-A antibody) on 10 patients with MSI mCRC showed an overall response rate (ORR) of 30%, and the rate of disease control was 90% without unanticipated toxicity [135]. Durvalumab is a human IgG1 mAb against PD-L1 that binds to the PD-L1 receptor and prevents interaction between PD-1 and PD-L1 [136]. The efficacy and safety of Durvalumab in monotherapy form was investigated in MSI-H tumors with 10 mg/kg IV administration every 2 weeks for 12 months. This trial showed 23% ORR for patients with MSI-H tumors and 22% ORR for patients with CRC. These results suggested Durvalumab as a promising treatment option for MSI-H tumors [137]. Avelumab is another fully human IgG1 mAb that binds PD-L1 and blocks the interaction between PD-L1 and its receptors, thus resulting in the restoration of immune responses, including T cell anti-tumor immune response. A study to evaluate the effective dose of Avelumab in 53 patients with metastatic or locally advanced previously treated solid tumors like CRC showed that the drug could be administrated in 20 mg/kg doses every 2 weeks, but further investigations are ongoing [138]. PD-L2 is another known ligand for PD-1 that is expressed in approximately 40% of CRC patients. The increased expression of PD-L2 in CRC is related to IFNγ expression and glycosylation [139]. Moreover, PD-L2 can influence the invasion of tumor cells. Thus, PD-L2 can be considered a new candidate for CRC treatment [140].

### 5.4. Anti-LAG-3

LAG-3 is an inhibitory immune checkpoint that has a significant role in maintaining immune homeostasis, reducing T cell proliferation, and inhibiting cytokine secretion [86]. Additionally, the co-expression of LAG-3 with PD-1 is a marker for CD8^+^ T cell exhaustion [141]. Accordingly, blockade of LAG-3 along with other negative immune checkpoints is an interesting therapeutic option for the promotion of antitumor immune responses [80,141]. Relatlimab is the first anti-LAG-3 fully human IgG4 mAb that was investigated as a therapeutic agent in multi-solid tumors such as CRC [142]. The results of a phase II study that was conducted to evaluate Relatlimab combination with Nivolumab (anti-PD-1) were associated with antitumor response in metastatic melanoma, and further studies are ongoing (NCT03743766) [143]. LAG525 and MK-4280 are other fully human IgG4 anti-LAG-3 mAbs that are currently in clinical trials. LAG525, in combination with anti-PD-1, is undergoing a phase I/II study in patients with a variety of advanced solid tumors and hematologic malignancies. The preliminary results of these investigations suggest a promising antitumor effect in neuroendocrine tumor (NET), small-cell lung cancer (SCLC), and diffuse large B-cell lymphoma (DLBCL) cancers [79,144,145]. MK-4280, as an anti-LAG-3 mAb, is currently under investigation in a phase I/II trial (NCT03598608) in combination with Pembrolizumab in patients with hematologic malignancies like classic Hodgkin’s lymphoma (cHL) and DLBCL [146]. Recently, in another study, LBL-007, a new anti-LAG-3 IgG4 antibody, was assessed in mice with CRC at a dose of 10 mg/kg twice a week for three weeks; it was found to inhibit tumor growth. Moreover, this study evaluated the combined effect of LBL-007 and anti-PD-1 antibodies, which can open new paths for further research on solid tumors [147]. Due to the lack of striking clinical studies done on anti-LAG-3 in CRC patients, further research and evaluations are required in this field.

### 5.5. Anti-TIM-3

TIM-3 (T cell immunoglobulin and mucin domain-containing protein 3), as an inhibitory receptor on T cells’ surfaces, helps the maintenance of immune hemostasis by providing peripheral tolerance [95]. This immune checkpoint induces apoptosis by binding to its ligand, galectin-9, and in this way, it can modulate T cell responses. A high expression of galectin-9 is detected in many solid tumors such as prostate cancer, cervical cancer, and melanoma [93]. It is significant to note that the available anti-murine and anti-human TIM-3 antibodies that have shown functional efficacy bind to TIM-3 in a manner that interferes with its binding to PtdSer and the adhesion molecule CEACAM1, and they have no role in the binding to galectin-9 [148]. MGB453 and TSR-022 are two IgG4 human anti-TIM-3 mAbs that are being investigated alone and in combination with anti-PD-1 mAbs in various advanced malignancies [149]. For instance, a recent phase I/II study evaluated the efficacy of MBG453 individually and in mixture with Spartalizumab (anti-PD-1 mAb) in patients with advanced malignancies, including CRC. The results demonstrated well-tolerable effects with the antitumor activity of the mixture MBG453 plus spartalizumab [150]. The overexpression of TIM-3 on CD8^+^ T cells in CRC patients could limit T cell activity, and blocking TIM-3 in these patients might promote T cell anti-tumor immune response [100]. Overall, these studies provide sufficient evidence supporting anti-TIM-3 mAbs as candidates for further research into CRC treatment, either alone or in combination with other ICIs.

### 5.6. Double Blockade of Immune Checkpoints

Despite many advances in cancer treatment using immune checkpoints, the efficacy of this method in some cancers, such as CRC, has been less evident than in others [151,152]. Moreover, it has been observed that blocking one inhibitory immune checkpoint is correlated with the upregulation of other inhibitory immune checkpoints, which neutralizes the therapeutic effects of mAbs and increases resistance to therapeutic approaches [153]. Therefore, combination therapy targeting multiple immune checkpoints simultaneously obtains better results for developing optimal therapeutic approaches [149,152]. A combination of anti-CTLA-4 with anti-PD-1/PD-L1 was found to demonstrate a synergistic effect in melanoma, RCC, and mCRC patients with MMR/MSI-H [53]. A co-blockade with anti-CTLA-4 (Ipilimumab) and anti-PD-1 (Nivolumab) has shown effectiveness and is FDA-approved in patients with dMMR/MSI-H mCRC [112]. In preclinical experiments on a murine colon cancer model (CT-26), a double blockade of CTLA-4 and PD-L1 enhanced tumor rejection and completely inhibited liver metastasis, while blocking CTLA-4 or PD-L1 alone caused a decrease in liver metastasis. Notably, this study showed that blocking CTLA-4 in combination with PD-L1 promotes intratumoral CD8^+^ and CD4^+^ T cells and decreases Treg cells. Another significant result was that the dual blockade of CTLA-4 and PD-L1 increased the expression of IFN-γ, IL-1α, IL-2, and IL-12 cytokines [152]. A study reported that the MSI subset of CRC had not expected response to treatment with PD-1 blocking; therefore, combination immunotherapy with checkpoints can be a suitable approach for treating this CRC subset [154]. In line with these findings, a combination of Nivolumab plus Ipilimumab displayed a good response in MSI-H/dMMR with mCRC. The outcomes showed a 55% ORR in 12-month with an OS rate of 85% in 119 patients who received Nivolumab (3 mg/kg) combined with Ipilimumab (1 mg/kg) every 3 weeks [115]. Similar evidence was found in a phase II study that combined Durvalumab (anti-PD-L1) and Tremelimumab (anti-CTLA-4), obtaining an improvement of overall survival in patients with advanced refractory CRC [120]. In evaluating the combined effect of other checkpoints, the combination of anti-LAG-3 and anti-PD-1 had a promising result in treating solid tumors. MK-4280 mAb plus Pembrolizumab, as another research direction in this field, is currently in phase I/II trial on hematologic malignancies [155]. The use of anti-TIM-3 and other ICIs, along with anti-PD-1 mAbs, may have promising outcomes in patients [153]. Therefore, the combination of anti-PD-1/PD-L1 and anti-TIM-3 as a suitable treatment approach for other studies can be considered. Currently, several studies are clinically examining agents that individually or combinatorically block TIM-3 and PD-L1, including combinations of LY3321367 as anti-TIM-3 with LY3300054 as anti-PD-L1 mAbs in patients with advanced solid tumors [156].

## 6. Combination of Immune Checkpoint Inhibitors with Other Immunotherapies

In addition to immune checkpoint inhibition, multiform immunotherapies with various targets could be effective against cancer. To make new cancer therapy more effective, the combination of ICIs with other potential immunotherapy methods such as cancer vaccines, oncolytic viruses, adoptive T cell therapy, and targeted therapy using small-molecule inhibitors are appropriate approaches for enhancing antitumor immune response [157,158]. Cancer vaccines are essential in the stimulation of presenting tumor-associated antigens (TAAs) by APCs. Therefore, the use of cancer vaccines plus ICIs may exert a synergistic antitumor effect [111]. For instance, a preclinical study examined the effect of anti-PD-1 and granulocyte-macrophage colony-stimulating factor (GM-CSF) in mouse models of colon cancer; the results showed that the combination of anti-PD-1 and GM-CSF significantly increased the antitumor response and enhanced survival [159]. Additionally, anti-PD-1 mAbs concomitant with GM-CSF enhanced T effector cells in the TME and increased the secretion levels of Th1 cytokines in colon and melanoma cancers [160].

Like cancer vaccines, oncolytic viruses have crucial roles in improving immune cells’ response against cancer cells. Recent preclinical studies have shown the antitumor effects of oncolytic viruses like oncolytic herpes simplex virus type 2 (oHSV2) and reovirus, respectively, in CRC and melanoma [161,162]. Oncolytic viruses in combination with anti-PD-1 modify the TME by increasing TILs and improving anti-PD-1 mAb function [163]. A combination of oncolytic adenovirus ONCOS-102 with Pembrolizumab demonstrated a synergistic antitumor effect in a melanoma mouse model. The intratumoral administration of talimogene laherparepvec (T-VEC) as an oncolytic virus combined with anti-PD-1 boosted antitumor response in patients with unresectable stage III–IV metastatic melanoma [164,165]. Another study that performed both in vitro and in vivo conditions in colon adenocarcinoma provided significant evidence based on the combination of Ad-CEA vaccination with anti-PD-1 mAb; this study detected augmented T-cell infiltration with a reduction in Tregs [166]. Moreover, oncolytic viruses and anti-PD-1 combination therapy can decrease the resistance of the TME in response to treatment in refractory cancers [167].

The combination of adoptive T cell therapy with ICIs is another strategy. Recently, adoptive T cells engineered to express chimeric antigen receptors (CARs) with tumor specificity have shown remarkable success in promoting tumor antigen recognition and enhancing antitumor responses by T cells [157]. CAR T cell therapy has exhibited efficacy in treating hematologic B cell malignancies, though there is insufficient evidence for the success of this treatment in solid tumors [168]. Though several studies have demonstrated a decrease in the efficiency of CAR T cells due to the immunosuppressive TME, recent evidence revealed that a blockade of PD-1 in combination with CAR T cells could increase antitumor effects against solid tumors. Therefore, the simultaneous utilization of PD-1 inhibition and CAR T cell therapy may demonstrate effectiveness in improving solid tumor treatment [169,170].

The combination therapy of ICIs with small molecules that target various pathways, including epidermal growth factor receptor (EGFR) inhibitors, vascular endothelial growth factor (VEGF) inhibitors, indoleamine 2,3-dioxygenase 1 (IDO1) inhibitors, and Bruton’s tyrosine kinase (BTK) inhibitors are under investigation as novel anti-cancer strategies [158,171]. Anti-EGFR antibodies are often used to treat NSCLC, and their antitumor activity has been confirmed by their ability to increase the number of CTLs and reducing Treg function [165]. In preclinical studies, the combination of ICIs, such as anti-PD-1/PD-L1, with EGFR blockers has been found to improve the efficacy of ICIs in NSCLC and CRC [172,173,174]. However, it is essential to state that further investigations are necessary for this combination therapy due to treatment-related adverse effects [172]. According to previous findings, tumor cells cause the generation of new blood vessels and angiogenesis. VEGF, one of the factors involved in angiogenesis, inhibits TIL trafficking into the TME and prevents the activation of T cells. Therefore, blocking VEGF or VEGFR could diminish metastasis and promote T cell immune response in combating tumor cells [175]. Anti-VEGF inhibitors are currently utilized as monotherapy or in combination with ICIs to treat RCC, glioblastoma, breast cancer, and mCRC [175,176,177,178]. A recent clinical study reported that a combination of Ipilimumab (anti-CTLA-4 mAb) and Bevacizumab (anti-VEGF mAb) exhibited promising activity with a predictable and manageable toxicity profile in glioblastoma [177]. Another study suggested that a combination of Atezolizumab (anti-PD-L1) with FOLFOX/Bevacizumab (anti-VEGF) may be beneficial as a treatment of unresectable mCRC without safety concerns; however, further investigations are needed to definitively assess the efficiency of this new approach [179]. IDO1 and BTK are other small molecules involved in tumor progression. IDO1 is an enzyme produced from various cell types, such as a small subset of plasmacytoid DCs in mouse tumor-draining lymph nodes, and it leads to tumor escape via the suppression of T cell activation in the TME [180,181]. Preclinical studies showed that IDO may lead to resistance to anti-CTLA-4 immune checkpoint therapy in hepatocellular carcinoma (HCC). Therefore, it is suggested that using IDO inhibitors combined with ICIs could improve the anti-cancer function of anti-CTLA-4 antibodies [182].

Similarly, a clinical study showed that the combination of Ipilimumab with epacadostat as an IDO inhibitor (≤50 mg) led to acceptable results with well-tolerated side effects in patients with unresectable or metastatic melanoma [171]. Ibrutinib is known as Bruton’s tyrosine kinase inhibitor with proven antitumor activity in different B-cell malignancies. Moreover, as a tyrosine kinase inhibitor, ibrutinib also targets interleukin-2-inducible kinase (ITK), an essential enzyme in T cell signaling. The blocking of ITK by ibrutinib affects Th2, not Th1, and shifts Th2 to Th1. Therefore, the use of ICIs plus ibrutinib could promote antitumor responses. The synergistic antitumor effect of anti-PD-L1 combined with ibrutinib has been indicated in mouse models of lymphoma and solid tumors, including breast cancer and colon cancer [183,184]. Nowadays, such combination trials are ongoing and further investigation is needed, but, overall, these combination approaches may be associated with improved outcomes in cancer patients.

## 7. Combination of Immune Checkpoint Inhibitors with Conventional Treatments

Immune checkpoint inhibitors have shown efficacy in various malignancies. Their combination with conventional therapies such as radiotherapy and chemotherapy could achieve better effectiveness than monotherapy [185].

### 7.1. Immune Checkpoint Inhibitors plus Radiotherapy

Radiotherapy is a conventional therapy that directly targets tumor cells and leads to immunogenic cell death in tumor cells [186]. Radiotherapy is an effective treatment that releases TAAs and induces the antitumor response by stimulating APCs [187]. Additionally, this method increases MHC I expression, which improves immune response [186]. The available evidence suggests that the combination of radiotherapy with ICIs has a synergistic effect and boosts antitumor response via releasing TAAs and cytokines, and it provokes T cells immunity against tumor cells [188]. For instance, a systematic review and meta-analysis study that compared the effect of ICI plus radiotherapy and ICI alone in CNS melanoma metastases, NSCLC, and prostate cancer models indicated the use of ICIs with radiotherapy as a safe approach and a good candidate for future clinical trials [189]. A preclinical study of the combination of radiotherapy (five daily fractions of 2Gy) with anti-PD-1 antibodies demonstrated a general systemic antitumor effect; it increased T cells responses in murine models of CT-26 and 4434 cell lines [190]. A study of a mouse model resistant to anti-PD-1 showed that radiotherapy induces IFN-β production, increases MHC I expression, and ultimately improves the immune response [191]. In a clinical study, the use of radiotherapy after Ipilimumab in patients with advanced melanoma indicated abscopal responses, which were associated with augmentation in overall survival [192]. In line with the previous result, the median survival in patients with melanoma brain metastases that had received Ipilimumab after radiotherapy was enhanced compared to patients that received Ipilimumab before radiotherapy. Accordingly, the definition of the optimal sequence of radiotherapy and ICIs may be vital for the combination therapy of radiotherapy and ICIs [193]. The selected dose for radiotherapy is also crucial for the effectiveness of the treatment and combination with ICIs. Overall, ICIs plus radiotherapy combination therapy has synergistic effects; still, more studies are required to confirm this approach.

### 7.2. Immune Checkpoint Inhibitors plus Chemotherapy

Chemotherapy is a common anti-cancer therapy that provides antitumor effects by enhancing tumor immunogenicity and inducing immunogenic cell death [194]. Cytotoxic chemotherapy eliminates cancer cells through various mechanisms, such as stopping DNA replication and transcription or destroying mitotic spindles [195]. Available evidence suggests that chemotherapy agents decrease circulating Tregs and MDSCs, thus promoting anti-cancer effects, and the combination of chemotherapy drugs with ICIs increases tumor cells’ sensitivity to ICI therapy [196,197,198]. The simultaneous use of chemotherapy with ICIs was evaluated in multiple solid tumors, particularly NSLCs and CRC [199,200,201,202]. For instance, the use of chemotherapy agents (ixabepilone and gemcitabine) combined with Ipilimumab showed a synergistic effect, reducing tumor growth in an animal model of CT-26 colon carcinoma [203]. Combining 5-fluorouracil plus oxaliplatin (FOLFOX) with anti-PD-1 led to successful tumor treatment in CRC mouse models. Additionally, the treatment of CRC patients with FOLFOX chemotherapy agent was found to lead to the high infiltration of CD8^+^ T cells into the TME and the expression of PD-L1, which is a suitable treatment method in combination with ICIs [199]. In line with these data, the FOLFOX agent was found to promote the efficacy of ICIs and to improve CD8^+^ T cells by improving exhaustion in CD8^+^ T cells in CRC [204]. The combination of decitabine and anti-PD-1 was found to inhibit the tumor growth and increase the survival of a CT-26 mouse model. Furthermore, the results indicated that decitabine improved the antitumor effect of the anti-PD-1 antibodies [200]. A preclinical study demonstrated that the combination of oxaliplatin with ICIs enhanced ICI therapy’s efficacy in a mouse model of CRC associated with an increased immune cell infiltration within TME [205]. Additionally, a study performed on a mouse model of breast and prostate cancer demonstrated that combining chemotherapy with ICIs reduces chemotherapy resistance [206]. Based on these promising findings, clinical trials are underway to investigate the combined effect of ICIs plus chemotherapy in several solid tumors. Table 1 reports the clinical trials of ICIs alone and in combination with other therapies for CRC.

## 8. Adverse Effects

Despite many achievements and advances in cancer treatment with ICIs, side effects are one of the challenges and limitations for ICI therapies. Adverse effects caused by ICIs are called immune-related adverse effects (irAEs), which are more common in organs such as the skin, gastrointestinal, lungs, kidneys, liver, and nervous system. According to available studies, the toxic effects of mAbs against CTLA-4 are more severe than anti-PD-1/PD-L1 antibodies due to their essential and comprehensive role in various subgroups of T cells (naive and memory) in lymph nodes [211]. Pruritus, rash, diarrhea, colitis, hepatic, hyperthyroidism, hypothyroidism, and pneumonitis are some of the adverse effects that follow the use of ICIs [212]. However, colitis is the most common irAE related to anti-CTLA-4 antibodies, while pneumonitis, hepatitis, and neurotoxic effects are usually associated with anti-PD-1/PD-L1 therapies [213]. It is worth mentioning that the irAEs of combination ICIs are much stronger than those of monotherapy [214]. In a case report study, it was found that the concurrent use of Ipilimumab and Nivolumab was correlated with more severe toxic epidermal necrolysis (TEN) than monotherapy in patients with metastatic melanoma [215].

Moreover, the combination of Nivolumab and Ipilimumab caused autoimmune myositis and myasthenia gravis in metastatic melanoma patients [216]. Overall, controlling and reducing these irAEs is one of the necessary aspects of treatment with ICIs. According to irAE grading (grades 1–4), various treatments such as corticosteroids can be considered [211].

## 9. Conclusions

CRC is classified as a group of solid tumors with high malignancy. In this respect, the malfunction of the TME in CRC is the major factor in homeostasis defection that provides an immunosuppressive microenvironment and contributes to tumor progression. Therefore, the targeting of immune checkpoints is an interesting new strategy for cancer therapy, with evidence for success in the treatment of malignancies such as melanoma and NSLC by amending the immune cells’ function in combating tumor cells. The blocking of immune checkpoints induces an extended durable response by preventing T cell exhaustion and promoting T cell antitumor response. Among well-known ICIs, anti-PD-1/PD-L1 and anti-CTLA-4 plus anti-PD-1/PD-L1 has shown remarkable responses in CRC. However, the results of several studies have demonstrated no considerable efficacy in these ICIs in CRC. Accordingly, the combination of ICIs together or with other therapeutic approaches has indicated promising results and might be a successful step forward in CRC therapy. However, preclinical and clinical data of ICI combinations in CRC are limited, and further studies are required to find appropriate approaches in the treatment of this cancer. On the other hand, the adverse effects of immunotherapy with ICIs and resistance to treatment in CRC patients are considered challenges for ICI immunotherapy in order to achieve better results. Regarding the importance of predictive immunotherapy biomarkers such as MSI and tumor mutational burden (TMB) in serving proper response to ICIs, analyzing these biomarkers before using any of mentioned immunotherapy agents could give rise to an increased chance of treatment efficacy. Consequently, further research will usher new hope in the CRC treatment pathway with ICIs by overcoming challenges. 

## Figures and Tables

**Table 1:** Clinical trials in colorectal cancer (CRC).

Target	mAbs	Patients	Phase	Trial	Ref
**CTLA-4**	Tremelimumab	mCRC	II	A study that showed no significant activity of Tremelimumab as monotherapy in refractory metastatic colorectal cancer patients.	[117]
**PD-1**	Nivolumab	dMMR/MSI-H mCRC	II	A study evaluating Nivolumab in colon cancer was associated with durable responses in patients with previous treatments.	[125]
**PD-1**	Pembrolizumab	MSI-H/MSS mCRC	I/II	An assessment of Pembrolizumab with napabucasin that showed antitumor effects with acceptable toxicities in mCRC patients.	[129]
**PD-1**	Pembrolizumab	MMRp CRC	II	A study to investigate efficacy of Pembrolizumab plus with GVAX/Cy showed no efficacy in mismatch repair proficient CRC.	[207]
**Anti-PD-L1**	Durvalumab	MSI-H CRC	II	An evaluation of the efficacy and safety of Durvalumab demonstrated a well-tolerable response in MSI-H CRC patients.	[137]
**Combination of Immune Checkpoint Inhibitors**					
**CTLA-4 and PD-1**	Ipilimumab and Nivolumab	dMMR/MSI-H mCRC	-	An assessment of Ipilimumab in combination with Nivolumab, which suggested of significant antitumor activity in dMMR/MSI-H mCRC.	[115]
**CTLA-4 and PD-L1**	Tremelimumab and Durvalumab	refractory CRC	II	An assessment of the efficacy of Tremelimumab and Durvalumab that showed enhanced overall survival (OS) in patients with advanced refractory CRC.	[208]
**Combination ICIs Plus Other Immunotherapy**					
**PD-L1**	Atezolizumab and cobimetinib	mCRC	III	An assessment of the antitumor effect and safety of combined Atezolizumab with cobimetinib and Atezolizumab monotherapy vs. regorafenib in patients with mCRC.	[209]
**PD-L1**	Atezolizumab and FOLFOXIRI/bevacizumab	mCRC	II	An evaluation of the efficacy of the combination of Atezolizumab with chemotherapy plus Bevacizumab in mCRC patients.	[179]
**Combination ICIs Plus Radiotherapy**					
**PD-1**	Pembrolizumab and Radiotherapy	Liver mCRC	Ib	An evaluation of the efficacy of the combination of stereotactic body radiotherapy for resectable liver oligometastatic in MSS/MMR proficient CRC.	[210]
**Combination ICIs Plus Chemotherapy**					
**PD-L1**	Atezolizumab and FOLFOXIRI/bevacizumab	mCRC	II	An evaluation of the effect of the combination of Atezolizumab with chemotherapy plus Bevacizumab in mCRC.	[179]

## Data Availability

Not available.
